# MEDIATING PROTEINS UNDERLYING THE RELATIONSHIP BETWEEN MITOCHONDRIAL DNA COPY NUMBER AND DEMENTIA FROM TWO COHORTS

**DOI:** 10.1093/geroni/igae098.4350

**Published:** 2024-12-31

**Authors:** Teresa Tian, David Zweibaum, Yong Qian, Luke Pilling, Jun Ding, Luigi Ferrucci

**Affiliations:** National Institute on Aging, Baltimore, Maryland, United States; National Institute on Aging, Baltimore, Maryland, United States; NIH, Baltimore, Maryland, United States; University of Exeter, Exeter, England, United Kingdom; National Institute on Aging, Baltimore, Maryland, United States; National Institute on Aging, Baltimore, Maryland, United States

## Abstract

Recent reports have shown that blood mitochondrial DNA copy number (mtDNAcn) is associated with dementia risk, but the biological underpinnings remain unclear. Identifying proteins that mediate this relationship may provide mechanistic insights. In the UK Biobank, we performed mediation analyses of 2,917 proteins via Olink platform in the association between mtDNAcn estimated using fastMitoCalc and future dementia diagnosis among 33,852 participants initially free of dementia (mean age=57 years). Over an average 13.2 years of follow-up, 891 participants (2.6%) developed all-cause dementia. We found 57 mediating proteins for all-cause dementia (FDR-p< 0.05), mostly from inflammation and cardiometabolic function panels and implicated in 24 pathways with top ranked pathways being TNFs binding to physiological receptors, coagulation system, plasma lipoprotein assembly/remodeling/clearance, degradation of the extracellular matrix, and activation of matrix metalloproteinases (p< 0.05). Three protein networks with more than 10 significant proteins were related to organismal injury and abnormality, hereditary disorders, cellular function and maintenance, post-translational modification, cell death and survival, and lipid metabolism. Of 57 mediating proteins for all-cause dementia, 4 proteins (TNFRSF11B, SMOC1, Fibronectin, and SMAD1) also mediated the relationship between mtDNAcn and cognitive impairment or dementia in the validation cohort of 593 Baltimore Longitudinal Study of Aging participants with available cross-sectional data (mean age=76.8 years, SomaScan, p< 0.05). These findings may suggest that proteins important for energy metabolism, neurotransmitter synthesis, lipid and glucose homeostasis, and immunity may underlie the relationship between mitochondrial dysfunction and dementia risk. Future longitudinal studies should further validate these findings and investigate longitudinal changes in proteins.

